# Bias Controller of Mach–Zehnder Modulator for Electro-Optic Analog-to-Digital Converter

**DOI:** 10.3390/mi10120800

**Published:** 2019-11-21

**Authors:** Shuxin Shi, Jun Yuan, Qin Huang, Chongyu Shi, Xin Luo, Shan Lu, Pengfei Yuan, Hua Yu, Qiuqin Yue

**Affiliations:** 1 Key Laboratory for Optoelectronic Technology &Systems, Ministry of Education of China, Key Laboratory of Fundamental Science of Micro/Nano-Device and System Technology, Chongqing University, Chongqing 400044, China; 20160802007t@cqu.edu.cn (S.S.); 20152521@cqu.edu.cn (Q.H.); 201708021004@cqu.edu.cn (C.S.); 201708131108@cqu.edu.cn (X.L.); 20103160@cqu.edu.cn (S.L.); yuanpf@cqu.edu.cn (P.Y.); 2 Science and Technology on Analog Integrated Circuit Laboratory, Chongqing 401332, China; yjwlw@semi.ac.cn; 3 Chongqing College of Electronic Engineering, Chongqing 401331, China

**Keywords:** electro-optic analog-to-digital converter (ADC), Mach–Zehnder (MZ) modulator, bias point controller, pilot tone-based method

## Abstract

As one of the core devices for an electro-optic analog-to-digital converter (ADC), the Mach–Zehnder (MZ) modulator plays an important role, and the output stability of the MZ modulator has a decisive influence on the conversion accuracy of the ADC. This paper proposed a pilot tone-based method to stabilize the bias point of the modulator. This method could obtain the corresponding control voltage of the MZ modulator by adding a KHz-level dither tone to the bias end of the modulator and calculating the ratio of the first and second harmonic components. The experimental results showed that the output optical power of the modulator was stable at 3.2 dB when the bias point of the modulator was set at the orthogonal point. Moreover, the fluctuation range was not more than 0.15 dB, the first harmonic of the output signal was stable at 50.5 dB, and the fluctuation range was not more than 0.6 dB. The proposed bias controller based on the field programmable gate array (FPGA) and digital signal processing (DSP) can stabilize the modulator bias point at the orthogonal point and with a relatively high locking accuracy.

## 1. Introduction

Owing to the influence of the aperture jitter of the sampling clock [1], electronic analog-to-digital converters (ADC) have not been well applied in the field of high-rate signal acquisition and processing. ADC based on the combination of optics and electronics, can realize high-precision analog-to-digital conversions for ultra-high-speed analog signals by utilizing the advantages of wide bandwidth, fast speed, and the high accuracy of optical information processing. As the core device for photoelectric conversion in electro-optic ADC, the output stability of the electro-optic modulator has a great influence on the conversion accuracy of the ADC. Lithium niobate (LN) Mach–Zehnder (MZ) modulators are often used in electro-optic ADC due to their low optical relative loss, high optical power processing capability, and wide optical bandwidth [2,3,4]. In the future, the MZ modulator should be integrated with a smaller size. Reed et al. discussed the techniques that have been and will be, used to implement silicon optical modulators, as well as the outlook for these devices, and candidate solutions for the future [5]. Wang et al. overcame the difficulty of integrating electro-optic modulators and demonstrated monolithically integrated lithium niobate electro-optic modulators that featured a complementary metal oxide semiconductor (CMOS)-compatible drive voltage, supported data rates up to 210 gigabits per second and showed an on-chip optical loss of less than 0.5 decibels [6]. Haffner et al. introduced a 70 GHz all-plasmonic Mach–Zehnder modulator that fitted into a silicon waveguide of 10 μm length, where the technology suggested a cheap co-integration with electronics [7].

However, the physical properties of the MZ modulator are easily affected by the change of external environment, which results in the drift of its bias point and affects the output performance of the electro-optic ADC [8]. Therefore, it is of great significance to study the optimal bias point control of the MZ modulator for the electro-optic ADC.

Sekine et al. proposed a method for controlling the bias point of an optoelectronic modulator based on the optical power method [9]. This technique has the advantage of being able to lock to any offset position, but it requires additional light sources in the system, which adds to the complexity of the system. Moreover, since the output optical power signal strongly depends on the input optical power fluctuation of the MZ modulator and the optical path loss variation, the control precision of the system is limited. In Reference [10], a bias point modulation scheme of the electro-optical modulator based on the principle of the pilot method was proposed. Although this method can control the bias point of the modulator, it is necessary to adjust the parameters when the input light intensity varies, which may increase the difficulty of use. In Reference [11], Hao et al. proposed a composite control algorithm based on the average optical power slope value and the cotangent value, which solved the shortcomings of the output optical power of the modulator affected by the input optical power. Nevertheless, it requires a large amount of calculation. As such, these schemes have their advantages, but there are still problems of low control precision, cumbersome control methods, or the need for complicated control algorithms. Therefore, it is very important to design an automatic bias control system with high control precision, easy operation, as well as a relatively simple control algorithm to achieve the stable operation of modulator.

The paper is organized as follows. Firstly, the architecture of the electro-optic ADC and the operating characteristics of the MZ modulator are analyzed. Secondly, the bias point controller of the MZ modulator based on the field programmable gate array (FPGA) and digital signal processing (DSP) is proposed. Then the working principle and mechanism are described. In the next section, the test experiment system is set up and implemented. Finally, the experimental results and conclusions are provided.

## 2. Theoretical Analysis

### 2.1. Bias Point Control of the Electro-Optic Modulator

The MZ modulation is a key part of the electro-optic ADC system. The MZ modulator realizes the intensity modulation of the carrier optical signal, and the relationship between the output intensity of the modulator and the applied electric field voltage is as follows [12]:

(1) $$P_{\mathrm{out}}=\frac{T_{D}P_{0}}{2}\left[1+\cos\left(\frac{\pi V}{V_{\pi }}+\varphi_{0}\right)\right]$$

where $P_{0}$ is the input power of the signal, $V_{\pi }$ is the half-wave voltage of the modulator [13], $T_{D}$ is the inherent loss of the modulator, and $\varphi_{0}$ is the inherent phase difference between two branch arms of the modulator.

**Figure 1:**
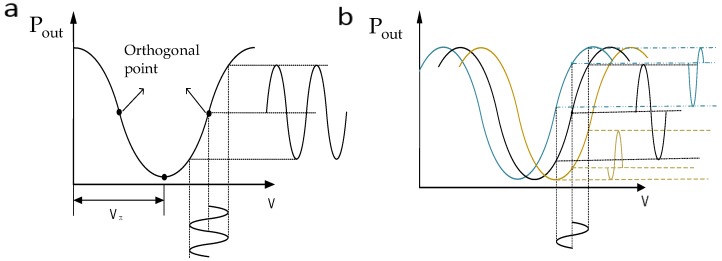
(**a**) Transmission characteristic curve of the Mach–Zehnder (MZ) modulator (**b**) Bias point drift curve of MZ modulator.

According to Formula (1), the intensity output characteristic curve of the MZ modulator can be obtained, as shown in Figure 1a. There are generally four commonly used bias points: positive and negative orthogonal points, maximum point, and minimum point [14]. The input signal can be directly modulated without distortion only when the bias point of the modulator is set in the linear region. Therefore, the bias point of the modulator is chosen to be set at the orthogonal point for the electro-optic ADC system.

The bias point of the MZ modulator is subject to external conditions. The external drift source includes the change of device temperature (thermooptic and thermoelectric effects), the change of optical power in the waveguide, and the strain relaxation of the silicon dioxide buffer layer in the device. These factors will lead to the change of the effective refractive index of the waveguide optical mode, which will cause the output characteristic curve of the modulator to move [15], as shown in Figure 1b. Once the external conditions change, the transmission characteristic curve moves to the left (blue curve) or right (yellow curve). When the voltage of the applied electric field is constant, the bias point of the modulator changes, which will change the waveform curve of the output modulated signal and affect the operation of the modulator system. Therefore, to achieve the best performance of the MZ modulator, the problem of bias drift must be solved.

### 2.2. Pilot Tone-Based Method for the Bias Point Controller

In the control scheme proposed in this paper, a dither tone with a frequency of KHz-level was added to the bias of the modulator to detect its first and second harmonic components to determine and control the bias point of the modulator [16].

Assuming that the input dither tone expression is  Vsin(ωt+θ(ω)), the phase shift expression of the optical signal caused by the dither tone needs to be changed to

(2) $$\Delta \emptyset =\frac{\pi V}{V_{\pi }\left(\omega \right)}sin\left(\omega t+\theta \left(\omega \right)\right)$$

where $V_{\pi }\left(\omega \right)$ is the half-wave voltage of the modulator as a function of frequency, and θ(ω) is the phase response delay of the modulated signal due to the mismatch of the microwave-optical speed. Substituting Equation (2) into Equation (1)

(3) $$P_{\mathrm{out}}=\frac{T_{D}P_{0}}{2}\left[1+cos\left(\frac{\pi V}{V_{\pi }\left(\omega \right)}sin\left(\omega t+\theta \left(\omega \right)\right)+\varphi_{0}\right)\right]$$

According to Reference [17], Equation (3) is first expanded by a trigonometric function and then expanded by the Taylor series. Thus, if we make $a=\pi V/V_{\pi }\left(\omega \right)$, Equation (4) can be obtained:

(4) $$P_{\mathrm{out}}=\frac{T_{D}P_{0}}{2}(1+cos\varphi_{0}(1-\frac{a^{2}}{4}+\frac{a^{4}}{64}+\left(\frac{a^{2}}{4}+\frac{a^{4}}{48}\right)cos\left(2\omega t+2\theta \left(\omega \right)\right)\;+\frac{a^{4}}{92}cos\left(4\omega t+4\theta \left(\omega \right)\right))\;-sin\varphi_{0}\left(\left(a-\frac{a^{3}}{8}\right)\cdot sin\left(\omega t+\theta \left(\omega \right)\right)\times \frac{a^{3}}{24}sin\left(3\omega t+3\theta \left(\omega \right)\right)\right)$$

From Equation (4), we can get the first harmonic component $I_{1st}$ and the second harmonic component $I_{2nd}$ of the corresponding photodiode output current and their harmonic ratio K,

(5) $$I_{1\mathrm{st}}=R\frac{T_{D}P_{0}}{2}sin\varphi_{0}\left(\frac{a^{3}}{8}-a\right)$$

(6) $$I_{2\mathrm{nd}}=R\frac{T_{D}P_{0}}{2}cos\varphi_{0}\left(\frac{a^{2}}{4}-\frac{a^{4}}{48}\right)$$

(7) $$K=\frac{I_{1\mathrm{st}}}{I_{2\mathrm{nd}}}=\frac{sin\varphi_{0}\left(\frac{a^{3}}{8}-a\right)}{cos\varphi_{0}\left(\frac{a^{2}}{4}-\frac{a^{4}}{48}\right)}=tan\varphi_{0}\frac{\left(\frac{a^{3}}{8}-a\right)}{\left(\frac{a^{2}}{4}-\frac{a^{4}}{48}\right)}$$

where R represents the responsivity of the photodetector. It can be seen from Equation (6) that the harmonic ratio R of the output signal is related to the bias voltage of the modulator for a given MZ modulator and a fixed input signal.

**Figure 2:**
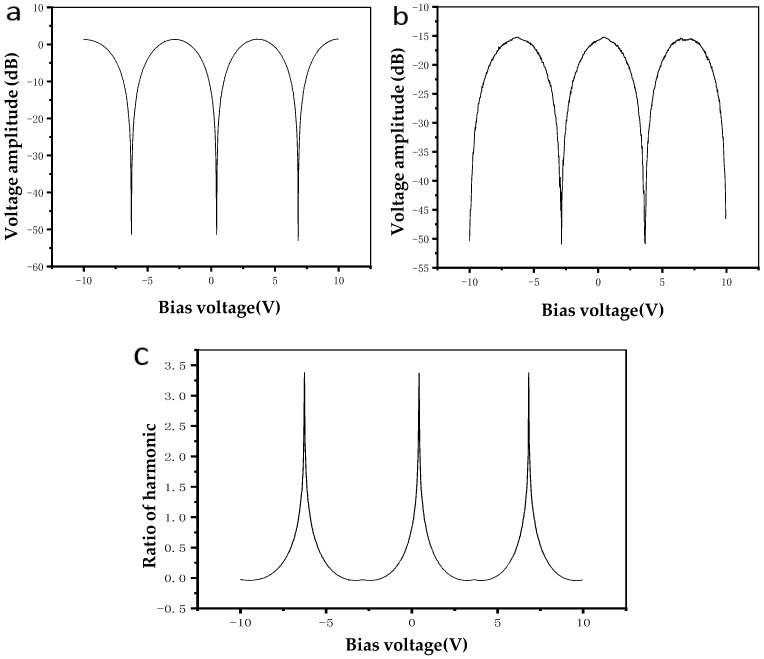
(**a**) Amplitude curve of the first harmonic of the dither tone. (**b**) Amplitude curve of the second harmonic of the dither tone. (**c**) The ratio of the first and the second harmonic.

Experiments were done to verify the theory of the control scheme. The amplitudes of the first and second harmonic components recorded were plotted, and their waveforms varying with bias voltage are shown in Figure 2a,b. The waveforms of their ratios varying with bias voltage are shown in Figure 2c.

It can be seen from Figure 2c that the curve of the first and second harmonic ratio changing with the bias voltage is approximately a tangent curve. By setting its positive and negative, it can be obtained that the ratio of the first and second harmonic is monotonically increasing with the bias voltage in a voltage cycle. Compared with Equation (6), it can be found that it is slightly different at the lowest point of the curve (the orthogonal working point of the modulator). Theoretically the curve at this point reaches the minimum value and shows a peak form, while the test curve is a relatively gentle curve. This result is because the first harmonic reaches the maximum value, and the second harmonic reaches the minimum value in theory at these two working points, so the ratio also approaches to 0. However, due to the limited accuracy of the test, it is not necessarily able to accurately capture the positive intersection point, only to collect some points with a very small range, so the measured data is not a peak, but a relatively gentle curve.

## 3. The Proposed Bias Point Controller of the MZ Modulator

### 3.1. Hardware Design of the Bias Point Controller

**Figure 3:**
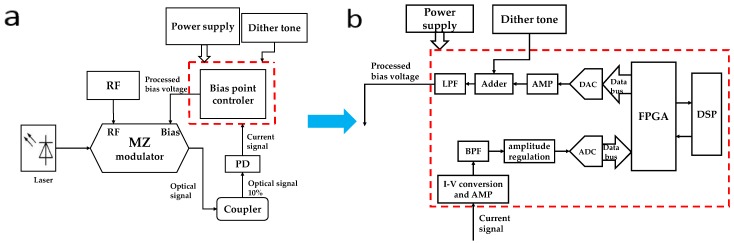
(**a**) Bias point controller architecture of the MZ electro-optic modulator (**b**) Detailed diagram of the bias point controller. Notes: PD, photodetector; AMP, amplifier; BPF, band-pass filter; LPF, low-pass filter

According to the application requirement of the MZ modulator in the electro-optic ADC system, the hardware design of the bias point controller was designed as shown in Figure 3a,b.

A coupler first separates the optical signal from the MZ electro-optic modulator and 10% of the optical signal is input to the photodetector. Then the photodetector converts this signal into a current signal. The current signal is very weak, so a trans-impedance amplifier with a very low bias current is needed to convert the current signal into a voltage signal. Then the voltage signal will pass through the two-stage amplifier circuit and the band-pass filter circuit to obtain a relatively pure modulated dither tone. After the signal amplitude adjustment circuit matches the amplitude of the signal with the input range of the ADC circuit, the analog signal will be converted to a digital signal and then inputted to the FPGA for spectrum conversion and processing.

In the FPGA, the frequency spectrum information of the disturbance signal is obtained using the fast Fourier transform (FFT) algorithm, and then the spectrum value of the second harmonic signal is found in the spectrum information. The value will be sent to the DSP for comparison. By comparing the obtained harmonic ratio with the harmonic ratio at the predetermined bias point, the required control voltage can be obtained.

In addition, the voltage corresponding to the harmonic ratio is stored in the DSP in the form of a database, which is obtained using a lookup table. The DSP transmits the control voltage value to the D/A converter through the FPGA and then superimposes the disturbance signal through the adder circuit. After passing through the low-pass filter, the signal is inputted to the DC bias terminal of the modulator to control the bias point of the modulator.

Finally, in the process of designing the circuit board, to ensure as much as possible that the analog signal is not interfered by the digital signal and the power signal on the circuit board, it is necessary that some effectual measures should be taken to separate the analog circuit and the digital circuit from the power supply circuit. At the same time, the analog signal should be separated from the digital signal by copper. A resistor of zero ohms connects the different ground signals.

### 3.2. Software Design of the Bias Point Controller

**Figure 4:**
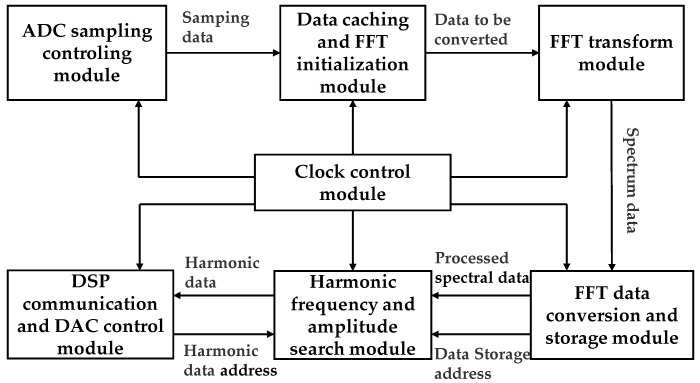
The overall architecture of the software.

The system adopts the processing structure based on the DSP and FPGA, and the FPGA completes the data acquisition, spectrum conversion, and first harmonic and second harmonic frequency amplitude search. The processing architecture block diagram is shown in Figure 4.

**Figure 5:**
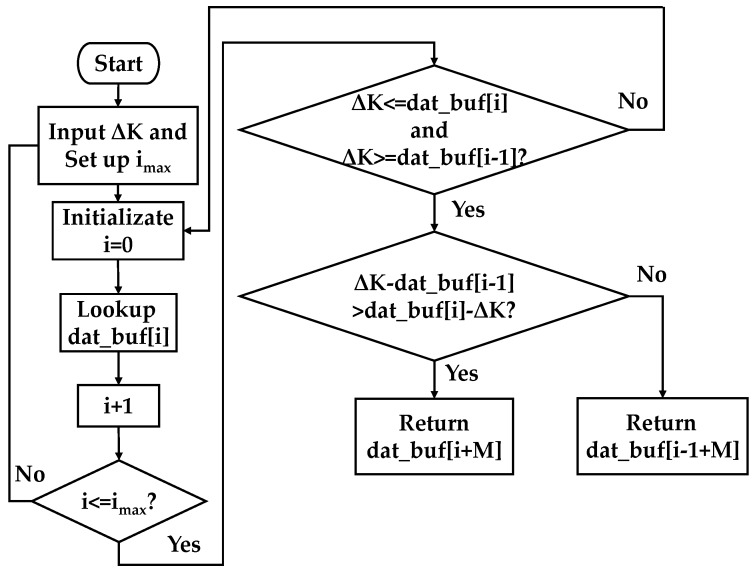
The algorithm flowchart to determine the control voltage.

The DSP completes the functions of data storage of the harmonic ratio at the work point, the division algorithm of first harmonic and second harmonic frequency data. The control algorithm flowchart is shown in Figure 5. ΔK denotes the difference between the harmonic ratios of two points, i is an addressing parameter, i_max_ is the maximum of the addressing parameters, and dat_buf is the value corresponding to the difference of the harmonic ratio.

After receiving the data from the FPGA, the DSP first divides the two harmonic data, obtains the ratio, and then subtracts the harmonic ratio of the orthogonal point, followed by comparing it with the data in the lookup table to find the closest one. The value is the control voltage that needs to be inputted to the bias point of the modulator.

## 4. Experimental and Results Analysis

**Figure 6:**
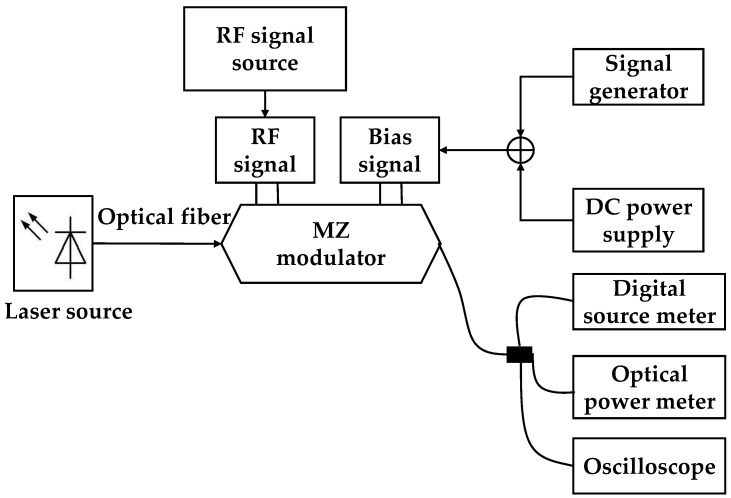
The principle block diagram of the test platform.

**Figure 7:**
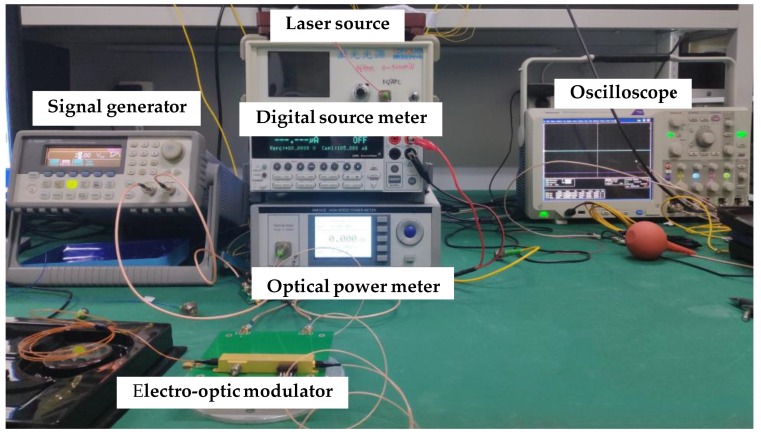
The photo of the test platform.

As shown in Figure 6, the principle block diagram of the test platform mainly includes a laser source, an optical fiber, an RF source, an MZ modulator, a signal generator, an oscilloscope, a digital source meter, and an optical power meter. Figure 7 is a picture of the experimental test system.

**Figure 8:**
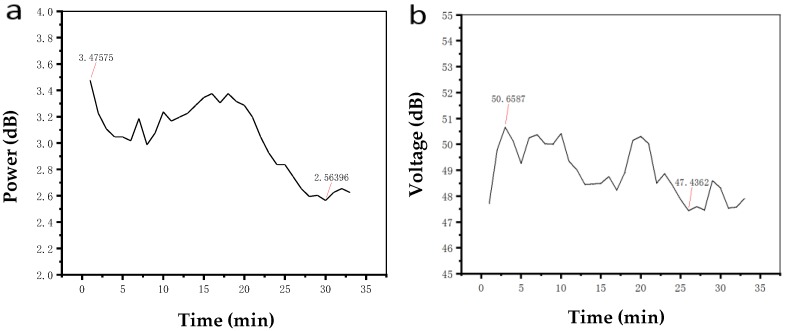
(**a**) Output optical power at the orthogonal bias points without the controller (**b**) First harmonic value at the orthogonal bias point without the controller.

Since the temperature has the greatest influence on the drift of the modulator bias point, we used the air conditioner to change the room temperature to increase the drift of the modulator bias point. Firstly, the output of the modulator without the control system was tested. The bias point of the modulator was set at the orthogonal point. By changing the operating temperature of the modulator, the output optical power of the modulator at the orthogonal bias point was observed for some time. The result is shown in Figure 8a. The value of the first harmonic component of the output signal was also recorded, and the result is shown in Figure 8b.

It can be seen from Figure 8 that the output optical power of the modulator changed greatly in 30 min. Its fluctuation range was between 2.5 dB and 3.5 dB, the upper and lower floating range was over 0.9 dB, and the fluctuation range of the first harmonic was more than 4 dB. It was obvious that the drift of the modulator’s bias point with time and external conditions was very significant.

**Figure 9:**
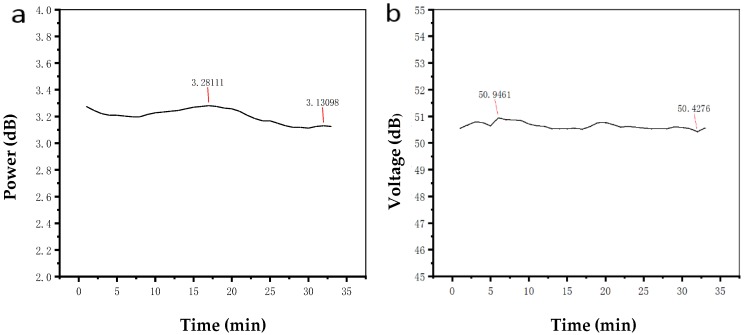
(**a**) Output optical power at the orthogonal bias point with the controller (**b**) First harmonic value at the orthogonal bias point with the controller.

Then, the control effect of the proposed bias point control system in this paper was tested. The input end of the controller connected to the photodetector and the output end of the controller was connected to the bias end of the MZ modulator. The output optical power and the first harmonic component of the modulator were recorded after 30 min with the change in external environment conditions. The results are shown in Figure 9a,b.

It can be seen from Figure 9a that the influence of time and environment conditions on the output optical power of the modulator becomes smaller when the control system is added. The output optical power of the modulator varied in the range of 3.13–3.28 dB, and the fluctuation range was about 0.15 dB. The first harmonic value of the output signal of the modulator varied between 50.42 and 50.95 dB, and the fluctuation range did not exceed 0.6 dB. The result showed that the proposed controller could better control the bias point of the modulator.

## 5. Conclusions

In this paper, a bias point controller of the MZ modulator based on FPGA and DSP was proposed for the MZ modulator in an electro-optic ADC system. The experimental results showed that the proposed controller in this paper could stabilize the bias point of the modulator at the orthogonal point. Compared with not using the controller, the stability of the output optical power of the modulator improved by at least four times. The research of the system has important engineering significance for the development of modulator control technology.
